# Chordoid Glioma With Dot-Like Immunoreactivity for Synaptophysin

**DOI:** 10.7759/cureus.13537

**Published:** 2021-02-24

**Authors:** Ashley Rose Scholl, Layla Nasr, Cesar A Serrano, Rudy J Castellani

**Affiliations:** 1 Department of Pathology, Anatomy and Laboratory Medicine, West Virginia University, Morgantown, USA; 2 Department of Radiology, West Virginia University, Morgantown, USA; 3 Department of Neurosurgery, West Virginia University, Morgantown, USA

**Keywords:** chordoid glioma, third ventricle, synaptophysin, contrast enhancement

## Abstract

Chordoid gliomas arise near the third ventricle and commonly present around 40 years of age. These rare tumors are non-invasive and often present with headaches and visual disturbances. Contrast enhancement on MRI is typical for these tumors and immunohistochemical (IHC) staining is positive for glial fibrillary acidic protein (GFAP). Surgical resection is the treatment of choice. We present this case of chordoid glioma because of its unique characteristics. The tumor lacked contrast enhancement on MRI and demonstrated juxtanuclear dot-like immunoreactivity for synaptophysin which is a feature not previously reported in the literature. It is important for pathologists and radiologists to be on the lookout for atypical presentations of these rare tumors.

## Introduction

Chordoid gliomas are rare, non-invasive tumors that usually arise within or near the third ventricle. These tumors were established as pathologic entities in 1998 and are classified as grade II by the 2016 World Health Organization (WHO) [1]. Even though they are classified as low-grade tumors, their location near hypothalamic structures can result in significant surgical morbidity and mortality secondary to the preferred treatment modality of surgical resection [2]. It has been suggested that these tumor's glial origins lie in the region of the lamina terminals, the circumventricular organs of the third ventricle, and the ependymal lining of this region [2].

The initial presentation of these tumors can vary from an incidental finding to an invasive at the time of diagnostic imaging [3]. There is a spectrum of presenting complaints, most commonly including intermittent headache and visual disturbances possibly due to increased intracranial pressure [1]. A recent literature review of 74 patients indicated a mean age at diagnosis of 43 years (range 5-71 years) and a female predominance of 2:1 [1,2]. Incompletely resected tumors can recur, but the overall prognosis for this tumor is good with close follow-up [2,3].

## Case presentation

**Figure 1:**
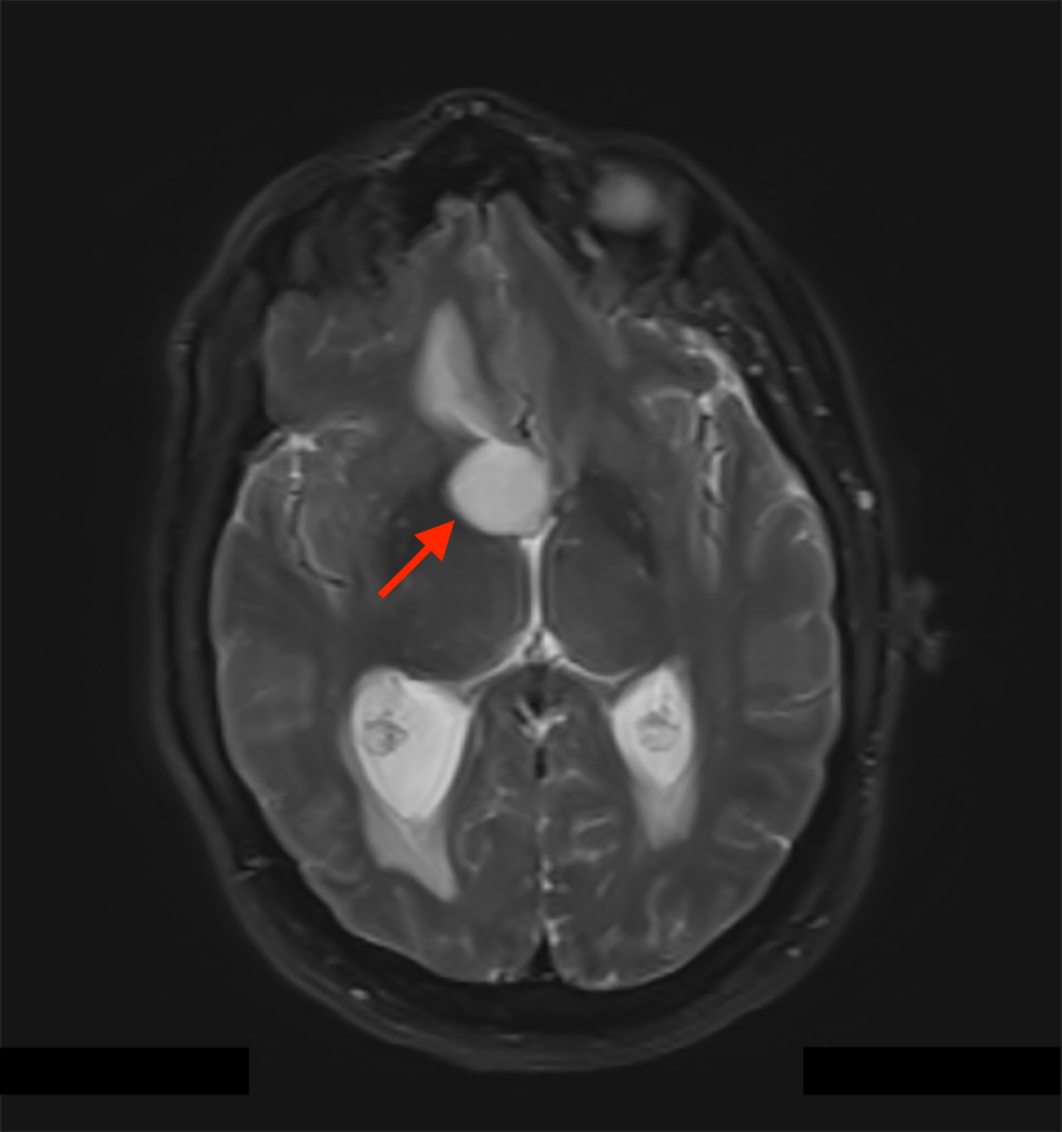
Axial T2 weighted MRI with the arrow indicating foramen of Monro. The lesion is a cystic-appearing intraventricular mass with associated obstructive hydrocephalus and no restricted diffusion.

**Figure 2:**
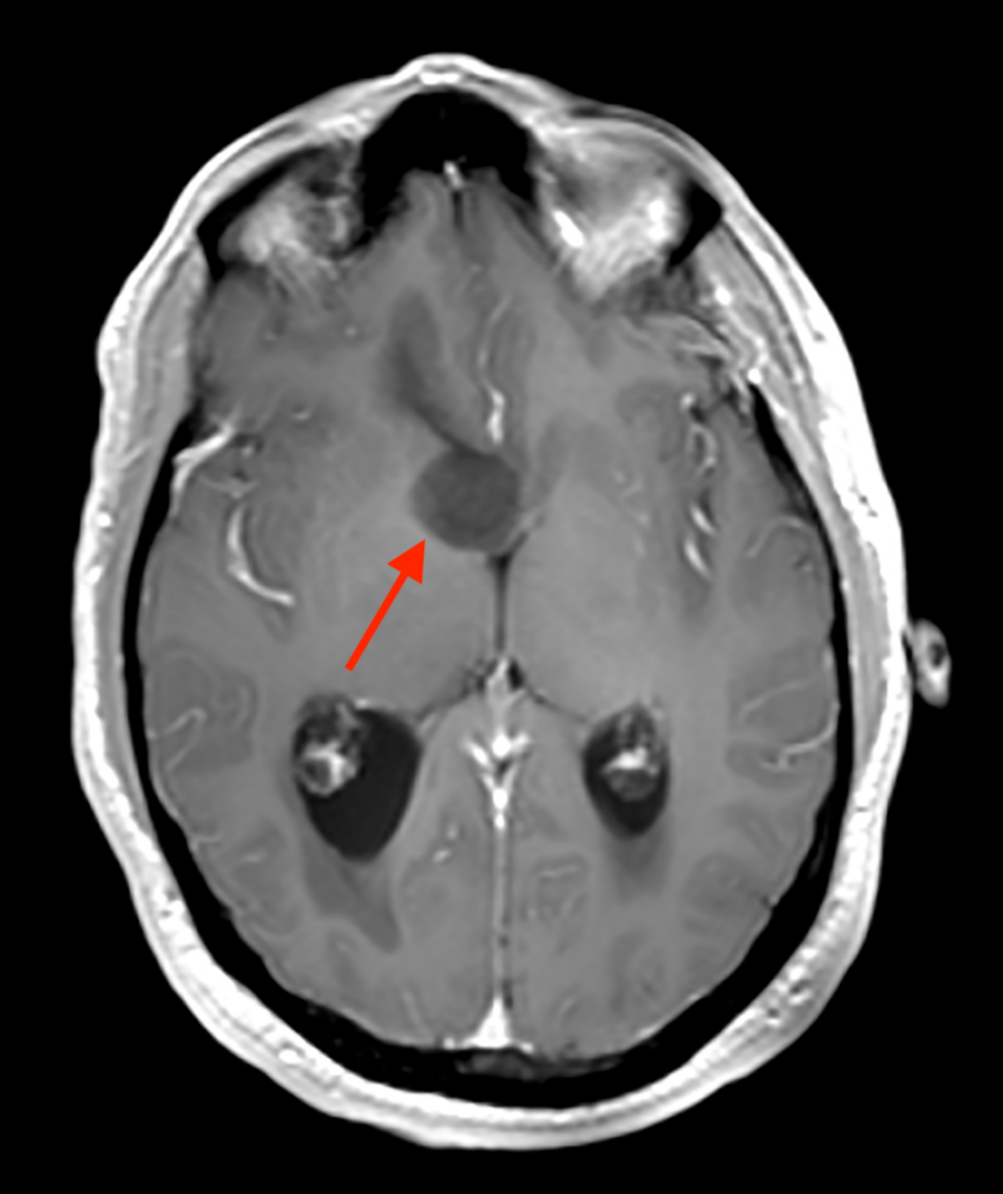
Post-contrast axial T1 weighted MRI with the arrow indicating foramen of Monro. The lesion is non-contrast enhancing, a feature not typically seen in this neoplasm.

**Figure 3:**
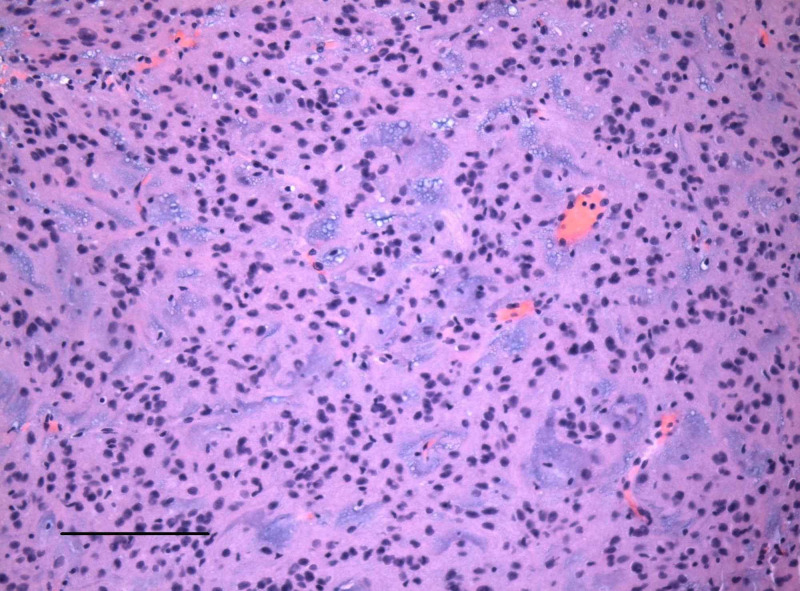
An H&E stained paraffin section shows cords of glial cells separated by a mucoid matrix and with focal cytoplasmic vacuolation (scale bar = 200 μm). The cells show mild cytologic pleomorphism, no discernible mitotic activity, and no microvascular proliferation or necrosis.

**Figure 4:**
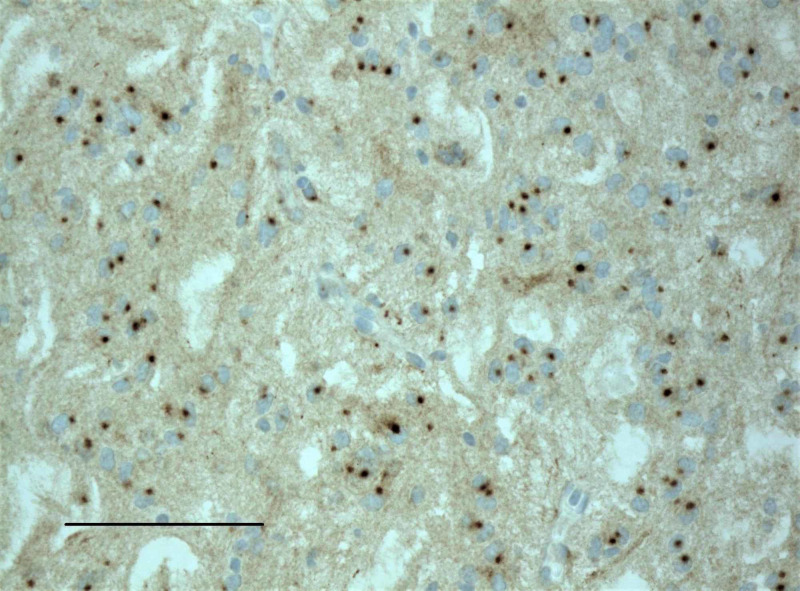
Immunohistochemistry for synaptophysin showed dot-like, juxtanuclear immunoreactivity (scale bar = 200 μm). This finding is previously undescribed in chordoid glioma.

**Figure 5:**
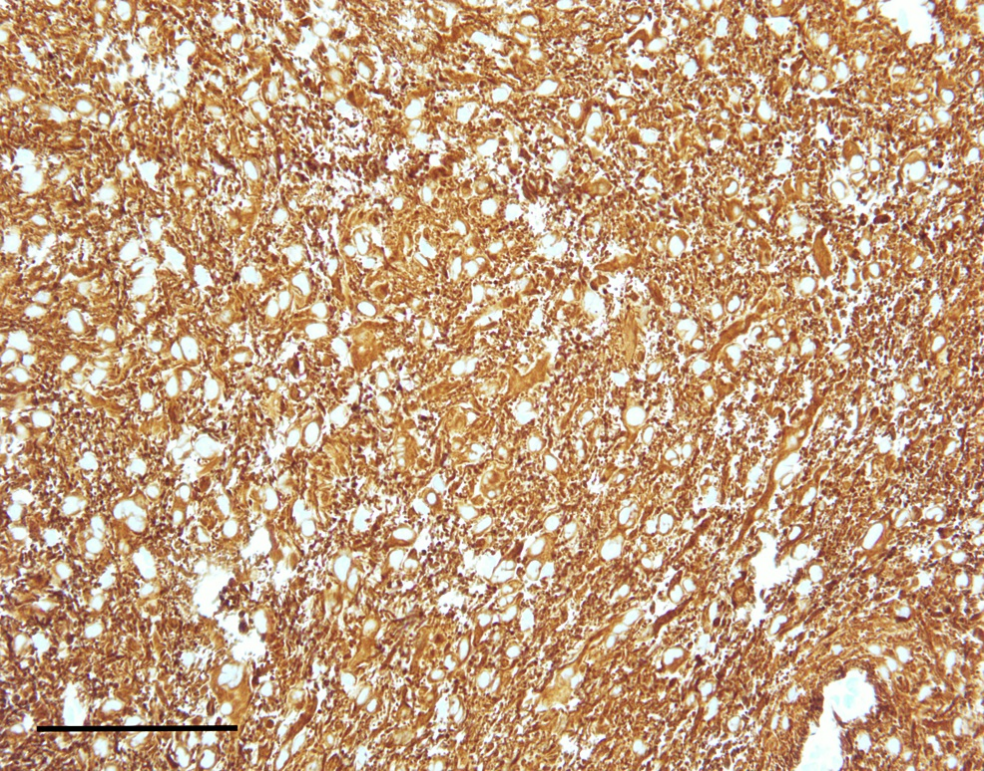
The lesion demonstrated strong immunoreactivity for glial fibrillary acidic protein (scale bar = 200 μm).

**Figure 6:**
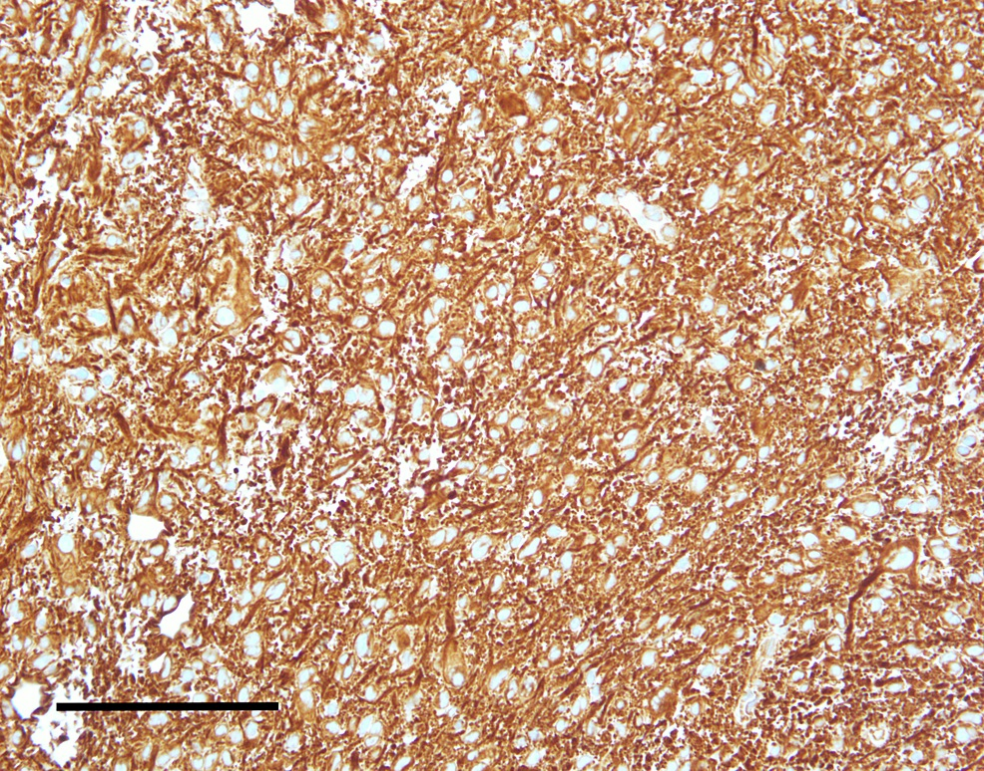
The lesion demonstrated strong immunoreactivity for vimentin (scale bar = 200 μm).

**Figure 7:**
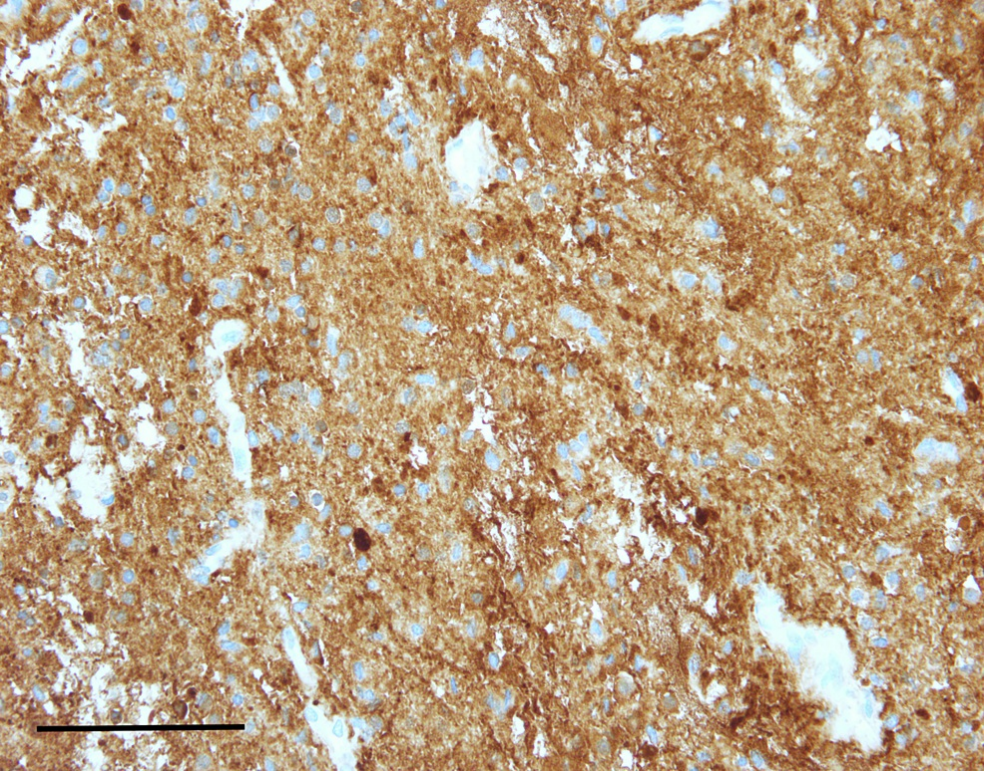
S100 showed patchy immunoreactivity (scale bar = 200 μm).

**Figure 8:**
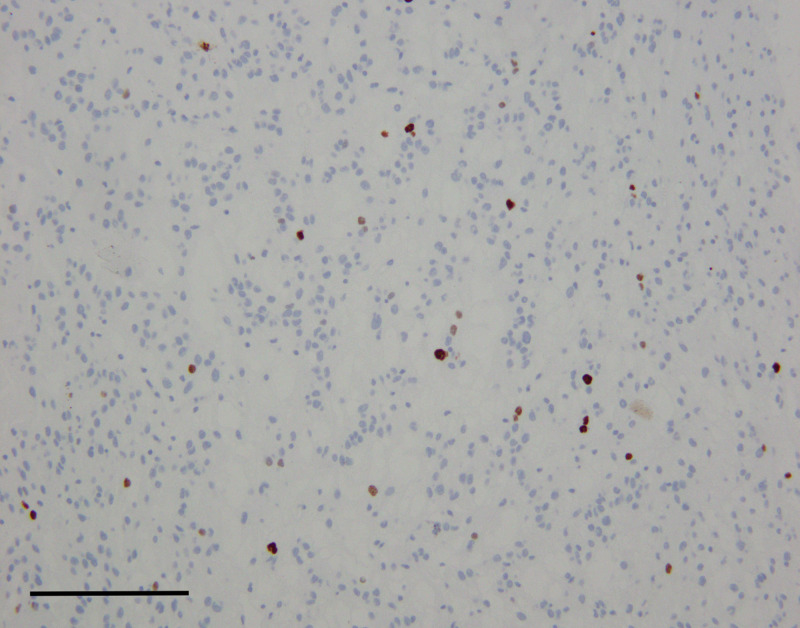
Ki-67 (Mib-1) showed a proliferation index of approximately 3% by visual inspection (scale bar = 200 μm).

A 48-year-old man with a past medical history of diabetes mellitus type II presented with a several-week history of acutely worsening ataxia, forgetfulness, nausea, intermittent vomiting, headache, and blurred vision. MRI showed a non-enhancing, cystic-appearing intraventricular mass centered on the third ventricle near the right foramen of Monro (arrow), with obstructive hydrocephalus and no restricted diffusion (Figures 1 and 2). A right frontal transcortical transventricular approach for resection of the tumor was performed, with post-operative imaging demonstrating a gross total resection. Pathological examination demonstrated morphologic features typical for chordoid glioma of the third ventricle (Figure 3), with some atypical immunohistochemical (IHC) findings including juxtanuclear dot-like reactivity for synaptophysin (Figure 4). Additional IHC findings include strong positivity for glial fibrillary acidic protein (GFAP; Figure 5) and vimentin (Figure 6), patchy positivity for S100 (Figure 7), and a Ki-67 of approximately 3% (Figure 8).

Differential diagnoses, in this case, include chordoma which was ruled out with positive IHC staining for GFAP. Additional differentials considered including meningioma which was ruled out with negative IHC staining for epithelial membrane antigen (EMA).

## Discussion

Chordoid gliomas typically demonstrate contrast enhancement by MRI [1], although our case lacked contrast enhancement. Histologically, chordoid gliomas are comprised of chords of GFAP-positive cells, often with cytoplasmic vacuoles resembling physaliferous cells of chordoma, in a mucinous extracellular matrix, as seen in this case (Figure 3). Immunoreactivity for CD34, EMA, and thyroid transcription factor 1 (TTF-1) is also variably reported. Interestingly, our case demonstrated juxtanuclear dot-like immunoreactivity for synaptophysin (Figure 4), a feature not previously reported. A recent analysis of 13 cases of choroid glioma demonstrated consistent D463H missense mutations in protein kinase C alpha (PRKCA), which localizes in the kinase domain of the encoded protein kinase C alpha [4]. The histogenesis of choroid gliomas is unclear, although one report indicated that choroid gliomas share TTF-1 expression with the organum vasculosum of the lamina terminalis, which suggests a circumventricular organ origin [5].

Chordoid gliomas are considered grade II by the 2016 WHO revised brain tumor classification, as tumors may recur following subtotal resection. Gross total resection, as achieved in this case, maybe curative. As such, surgical resection is the treatment of choice. At present, no evidence base exists for the role of adjuvant radiotherapy [1]. An apparent increased incidence of postoperative pulmonary embolism was reported by Nakajima et al. [6], the precise basis for which is unclear.

## Conclusions

We present a rare case of chordoid glioma of the third ventricle. Our case differed from those previously reported in that it lacked contrast enhancement on MRI and showed distinct, juxtanuclear dot-like immunoreactivity for synaptophysin. It will be interesting to see if subsequent cases are reported with these phenotypic features and whether these features correlate with a favorable outcome.
